# Normative data for rotational chair considering motion susceptibility

**DOI:** 10.3389/fneur.2022.978442

**Published:** 2022-08-22

**Authors:** Jiaodan Yu, Yi Wan, Jieli Zhao, Ruonan Huang, Peixia Wu, Wenyan Li

**Affiliations:** ^1^Ear Nose Throat (ENT) Institute and Department of Otorhinolaryngology, Eye and ENT Hospital, State Key Laboratory of Medical Neurobiology and Ministry of Education Frontiers Center for Brain Science, Fudan University, Shanghai, China; ^2^Nursing Department of Eye and ENT Hospital, Fudan University, Shanghai, China; ^3^Institutes of Biomedical Sciences, Fudan University, Shanghai, China; ^4^National Health Commission Key Laboratory of Hearing Medicine, Fudan University, Shanghai, China; ^5^The Institutes of Brain Science and the Collaborative Innovation Center for Brain Science, Fudan University, Shanghai, China

**Keywords:** sinusoidal harmonic acceleration test (SHAT), velocity step test (VST), visual suppression (VS), rotational chair test, motion sickness (MS), velocity storage mechanism (VSM)

## Abstract

**Objective:**

Rotational Chair Test (RCT) is considered one of the most critical measures for vestibular functionality, which generally includes the sinusoidal harmonic acceleration test (SHAT), velocity step test (VST), and visual suppression (VS). The purpose of this study was to establish normal values for different age groups on the RCT and investigate whether motion susceptibility, such as with a history of motion sickness or migraine, has any effects on test metrics.

**Methods:**

One hundred and nine subjects aged from 20 to 59 years who were free from neurotological and vestibular disorders were enrolled. According to the history of motion sickness or migraine, participants were divided into four groups: the motion sickness (MS) group (*n* = 13), the migraine group (*n* = 8), comorbidity group (*n* = 11), and the control group (*n* = 77). The 77 subjects without any history of MS and migraine were then further separated into four age groups: youth group (20–29 years), young and middle-aged group (30–39 years), middle-age group (40–49 years), and middle-age and elderly group (50–59 years). All participants underwent SHAT, VST, and VS, and a comprehensive set of metrics including gain, phase, asymmetry, time constant (TC), and Fixation Index were recorded.

**Results:**

Regarding the VST and VS, no significant differences were observed either across the four groups (MS, migraine, comorbidity, and control group) or four age categories within the control group. For SHAT, VOR gain at the frequency of 0.01 Hz, VOR phase from 0.08 to 0.64 Hz, and asymmetry at 0.01, 0.16, and 0.64 Hz indicated significant differences among various age groups (*P* < 0.05 for all comparisons). The VOR phase lead was lower in the migraine and comorbidity group than that in the control group at 0.64 Hz (*P* = 0.027, *P* = 0.003, respectively).

**Conclusions:**

Age slightly affects the result of SHAT, but not for VST and VS. VOR gain is more susceptible to aging at low frequency, while the phase is opposite. Subjects with both migraine and motion sickness show abnormal velocity storage mechanisms. Phase bias should be considered when assessing motion susceptibility with the RCT. SHAT is more sensitive than VST in terms of reflecting motion susceptibility.

## Introduction

It is well known that the peripheral vestibular system has frequency characteristics. The daily head movement frequency ranged from 0.05 to 6 Hz (1). Within this frequency range, the vestibular-ocular reflex (VOR) plays a vital role in maintaining visual stability. In past decades, videonystagmography (VNG) and the video head impulse test (v-HIT) have been commonly used to assess the vestibular system. But they both have their limitations. For example, the caloric test is equivalent to stimulating the vestibular system by adopting an aphysiological frequency range from 0.003 to 0.008 Hz. Moreover, it is unsuitable for the pediatric population and those with otologic surgery or meatal atresia (2). The v-HIT is a quantitative measure for assessing VOR with high-frequency (1–6 Hz) but is not adaptive for individuals with limited neck motion (1). The rotational chair test is complementary to the caloric test and v-HIT. The rotational chair test can test a broader range of frequencies from 0.01 to 0.64 Hz, provides a gold standard for diagnosis of bilateral vestibulopathy (BVP), and serves as a better choice for pediatric populations who cannot tolerate caloric irrigation test (3–5). Although the rotational chair test is less sensitive to acute unilateral peripheral disorders than the caloric test and v-HIT, it can assess vestibular compensatory status by analyzing parameters (1, 6). However, the normative data of the rotational chair test needs further study, and whether the parameters are age-related remains controversial.

Motion sickness (MS) is physiological vertigo commonly seen in the general population, but its susceptibility and severity vary between individuals. During exposure to vehicle motion or complicated and virtual vision, MS generally occurs with symptoms such as nausea, vomiting, and dizziness (7). The most cited pathogenesis for MS is the sensory conflict and neural mismatch hypothesis (8). Previous research reported that motion sickness and migraine are reciprocally connected. It is evident that migraine people increase motion sickness susceptibility (9, 10). Motion sickness susceptibility is related to VOR's spatial-temporality through activating the velocity storage mechanism (VSM) (11–13). In theory, motion sickness susceptibility generally shows lower phase lead and higher time constants (14). However, the empirical data are lacking. To fill that gap, this study aims to investigate what parameters of RCT differ in subjects with or without a history of motion sickness or migraine, or both.

The purposes of this study are divided into two parts. The first is establishing normal values for different age groups on the rotational chair test. Then, the second aim is to compare the control, MS, migraine and comorbidity groups, providing inspiration when assessing vestibular functionality by rotatory chair test.

## Materials and methods

### Subjects

This research study was conducted from June 2020 to March 2021. This study was approved by the Institute Review Board of Fudan University Eye Ear Nose and Throat Hospital (Reference Number 2020087). To create a representative sample of the general population, we recruited 109 subjects free from neurotological and vestibular disorders. Exclusion criteria included a history of otologic surgery or head injuries, cerebrovascular diseases, and systemic disorders. The subjects were asked to fill out a questionnaire about whether they had motion sickness and a history of migraine. Thirteen subjects reported having only motion sickness and 8 subjects had only a history of migraine. There were 11 patients who had both motion sickness and migraine. The remaining 77 subjects were assigned to four age groups: youth group (20–29 years), young and middle-aged group (30–39 years), middle-age group (40–49 years), and middle-age and elderly group (50–59 years). All subjects were told to refrain from eating for 2 h before the testing.

### Rotational chair test

A rotational chair test was performed using the VertiChair (ZT-CHAIR-I, Shanghai ZEHNIT Medical Technology Co., Ltd., Shanghai, China). Two technicians performed all the tests using a standardized protocol available before this study's start. The whole rotational chair system can implement a sinusoidal harmonic acceleration test, velocity step test, and visual suppression. The subjects were seated in the rotational chair with a safety belt in the completely dark room. The goggles were placed on the subject's faces and were tightened to the head with an elastic band to avoid slipping. The head was restrained and tilted forward 30° so that horizontal semicircular canals could be stimulated effectively. The subjects were told to keep alert and their eyes open throughout the whole testing procedure. When necessary, the operating technician communicates verbally to help the subjects maintain a constant awareness.

#### Sinusoidal harmonic acceleration test

The chair was rotated with sinusoidal waves at various range of frequencies (0.01, 0.02, 0.04, 0.08, 0.16, 0.32, and 0.64 Hz). The maximum velocity is 50 degrees/s. During the sinusoid rotation, the primary outcome variables were gain, phase, and asymmetry. The gain is calculated by dividing the eyes' slow component velocity by the chair's velocity. It can't orientate the vestibular lesion side but reflect the system's overall responsiveness. The phase reflects the temporal relationship between head movement and eye movement. It's called phase lead, reflecting the eye movements lead to head movement. If eye trace is ahead of head trace, phase lead will be a negative value. Otherwise, it will get a positive phase value called phase lag. The asymmetry reflects the difference in intensity of nystagmus of leftwards and rightwards directions.


$$\begin{array}{l} y_{\text{eye}}=A_{\text{eye}}\;*\;\sin\left(\omega_{\text{eye}}t-\varphi_{\text{eye}}\right)\\ y_{\text{head}}=A_{\text{head}}\;*\;\sin\left(\omega_{\text{head}}t-\varphi_{\text{head}}\right)\\ \text{phase}=\varphi_{\text{eye}}-\varphi_{\text{head}} \end{array}$$

*y*: the real-time speed of the fitted sine curve; A: the maximum speed (°/s); ω: rotational angular velocity; φ: the initial phase shift in degree.


$$\begin{array}{l} \text{Asymmetry}=\left(\text{a }-\text{ b}\right)/\left(\text{a }+\text{ b}\right)\times 100\% \end{array}$$

a: maximum right-beating slow phase average eye velocities; b: maximum left-beating slow phase eye velocities.

#### Velocity step test

Firstly, the chair has attained a velocity (90 degrees/s) with a constant acceleration (3 degrees/s^2^) in a clockwise direction. Constant acceleration causes nystagmus to occur. Although the subject's whole body was rotated, the right horizontal semicircular canal was stimulated in the acceleration process. Then, the chair was operated at 90 degrees/s for 1 min. As the chair was rotated constantly, nystagmus decayed exponentially. Finally, the chair was decelerated to 0 degrees/s in 1 s. The nystagmus that we recorded occurred in the opposite direction. The rotation in the clockwise direction stopped suddenly was equal to stimulation of the left semicircular canal. The whole procedure was then repeated in a counterclockwise direction. Two parameters were also measured during the velocity step test. They were post-rotary gain and time constant (TC). The gain is the ratio of the eyes' maximum SPV to the chair's velocity. The TC is the time for the VOR response to decay 37% of the maximum SPV.

#### Visual suppression

The subjects were rotated sinusoidally at a single frequency for three cycles with a peak velocity of 50 degrees/s. After that, subjects were instructed to fixate on the laser dot that appears on the Googles. The average VOR gain with a laser dot was compared with that without a laser dot. The Fixation Index (FI) was calculated by dividing gain with laser dot by gain without laser dot and then multiplying 100.

### Statistical analysis

The IBM SPSS Statistics version 26.0 for Mac was used for statistical analysis. We first examined whether the data were normal distribution. Normality of quantitative variables was performed using the Shapiro-Wilk test. Data were described as mean, standard deviation (SD), Inter Quartile Range (IQR), or percentages. We examined whether the parameters were affected by gender by using a *t*-test. Four age groups were compared using a one-way analysis of variance (ANOVA). For not normally distributed variables, the Kruskal-Wallis test was used instead of the ANOVA. Pearson correlation coefficient was used to analyze the correlation between parameters of SHAT and age. Considering the effect of age on SHAT, multiple-factors ANOVA was applied to compare the difference in phase for four groups (motion sickness, migraine, comorbidity, and control). *Post-hoc* comparisons with Least-Significance-Difference Method (LSD) were used. One-way ANOVA was used to explore the difference between groups for VAT and VS. The *P*-value <0.05 was considered significant.

## Results

### Demographics of subjects

A total of 109 subjects aged from 20 to 59 years participated in this study. Thirteen out of 109 subjects had only motion sickness and 8 out of 109 subjects had only migraine. Eleven subjects with both motion sickness and migraine thus served as the comorbidity group. In the MS group, 10 subjects were female, and 3 were male. In the Migraine group, 6 subjects were female, and 2 were male. In the comorbidity group, 7 were female and 4 were male. The remaining 77 subjects were stratified by four age groups: 20–29 years (*n* = 22), 30–39 years (*n* = 13), 40–49 years (*n* = 24), 50–59 years (*n* = 18). The demographics of the subjects are detailed in Tables 1, 2.

**Table 1:** Demographics of the subjects.

**Age groups**	***N***	**Mean age ±SD**	**Sex [*****n*** **(%)]**	
			**Male**	**Female**
20–29	22	25.86 ± 2.47	9 (41)	13 (49)
30–39	13	33.77 ± 2.20	8 (62)	5 (38)
40–49	24	45.13 ± 3.22	11 (46)	13 (54)
50–59	18	54.06 ± 2.71	5 (28)	13 (72)
Total	77	39.79 ± 11.26	33 (43)	44 (57)

**Table 2:** Demographics of the four groups.

**Groups**	***n*/*N***	**Mean age ±SD**	**Male**	**Female**
Motion sickness	13/109	29.62 ± 9.32	3	10
Migraine	8/109	33.13 ± 10.32	2	6
Comorbidity	11/109	27.73 ± 4.54	4	7
Control	77/109	39.79 ± 11.26	33	44

*Comorbidity group indicated the subjects have both migraine and motion sickness*.

### SHAT

Three parameters of SHAT across the four age groups are shown in Table 3. The parameters at each frequency confirmed as normal distribution were compared with one-way ANOVA, whereas others were compared with the Kruskal-Wallis test. Gain of 0.01 Hz significantly differed across four age groups (*P* = 0.044). Phases of 0.08, 0.16, 0.32, and 0.64 Hz exhibited significant differences in four age groups (*P*_0.08_ = 0.019, *P*_0.16_ = 0.001, *P*_0.32_ < 0.001, *P*_0.64_ < 0.001, respectively). Asymmetry significantly differed at 0.01, 0.16, and 0.64 Hz among the various age groups (*P*_0.01_ = 0.031, *P*_0.16_ = 0.01, *P*_0.64_ = 0.005, respectively). No significant differences in gender were observed. There were mild correlations between age and three parameters. The gain at 0.01 and 0.02 Hz was positively related to the age (*r*_0.01_ = 0.334, *P*_0.01_ = 0.004; *r*_0.02_ = 0.248, *P*_0.02_ = 0.032). The phase values from 0.16 to 0.64 Hz was negatively correlated with age (*r*_0.16_ = −0.374, *P*_0.16_ = 0.001; *r*_0.32_ = −0.475, *P*_0.32_ < 0.001; *r*_0.64_ = −0.549, *P*_0.64_ < 0.001, respectively). Because the sign of phase indicated whether the phase was leading or lagging. The phase lead values from 0.16 to 0.64 Hz correlate with age clinically. The asymmetry at 0.01, 0.16, and 0.64 Hz was negatively correlated with age (*r*_0.01_ = −0.263, *P*_0.01_ = 0.023; *r*_0.16_ = −0.261, *P*_0.16_ = 0.025; *r*_0.64_ = −0.331, *P*_0.64_ = 0.004, respectively).

**Table 3:** SHAT normative values of four age groups.

**Frequency (Hz)**	**Parameters**	**Age groups (years)**				***P***
		**20–29**	**30–39**	**40–49**	**50–59**	
0.01	Gain	0.30 ± 0.08	0.27 ± 0.11	0.36 ± 0.14	0.38 ± 0.12	0.044^*^
	Phase	−44.86 ± 5.84	−45.08 ± 5.54	−41.62 ± 7.22	−43.81 ± 7.48	0.324
	Asymmetry	10.48 ± 10.78	12.46 ± 14.05	2.42 ± 12.16	4.19 ± 9.53	0.031^*^
0.02	Gain	0.37 ± 0.13	0.35 ± 0.14	0.42 ± 0.14	0.43 ± 0.12	0.189
	Phase	−26.86 ± 7.89	−24.69 ± 4.77	−21.87 ± 5.61	−24.20 ± 4.09	0.056
	Asymmetry	3.82 ± 9.45	3.69 ± 11.29	1.50 ± 12.36	−1.80 ± 7.13	0.392
0.04	Gain	0.44 ± 0.14	0.43 ± 0.14	0.47 ± 0.15	0.48 ± 0.12	0.395
	Phase	−11.95 ± 8.79	−13.00 ± 3.52	−13.46 ± 6.18	−14.50 ± 4.26	0.682
	**Asymmetry**	(−3.00, 6.50)	(−2.00, 18.00)	(−8.75, 9.75)	(−2.75, 8.00)	0.545
0.08	Gain	0.47 ± 0.15	0.41 ± 0.11	0.46 ± 0.17	0.54 ± 0.14	0.134
	Phase	−3.90 ± 4.92	−4.54 ± 4.43	−2.54 ± 7.09	−8.12 ± 4.29	0.019^*^
	Asymmetry	2.48 ± 10.23	3.23 ± 10.13	0.92 ± 9.82	−0.18 ± 8.52	0.749
0.16	Gain	0.47 ± 0.19	0.42 ± 0.11	0.47 ± 0.20	0.51 ± 0.15	0.614
	**Phase**	(−5.50, 4.00)	(−0.75, 9.50)	(−6.00, 2.00)	(−11.50, −1.75)	0.001^*^
	**Asymmetry**	(−4.00, 11.50)	(0.25, 15.50)	(−13.00, 8.00)	(−9.25, 5.00)	0.013^*^
0.32	Gain	0.54 ± 0.21	0.51 ± 0.17	0.48 ± 0.20	0.52 ± 0.19	0.539
	**Phase**	(−2.75, 2.75)	(−5.50, 5.00)	(−16.25, −0.75)	(−17.25, −5.50)	<0.001^*^
	**Asymmetry**	(−4.00, 6.75)	(−1.25, 8.50)	(−5.00, 7.00)	(−7.25, 3.25)	0.235
0.64	Gain	0.69 ± 0.19	0.62 ± 0.20	0.63 ± 0.21	0.69 ± 0.14	0.520
	**Phase**	(−8.00, 0.00)	(−6.50, 1.00)	(−29.00, −3.00)	(−37.00, −12.00)	<0.001^*^
	Asymmetry	3.81 ± 8.38	6.00 ± 6.71	−0.62 ± 6.83	−2.72 ± 7.14	0.005^*^

*Bold fonts indicate that data of parameters were presented as Inter Quartile Range*.

* *Statistically significant according to Kruskal-Wallis test or one-way ANOVA (p < 0.05)*.

**Figure 1:**
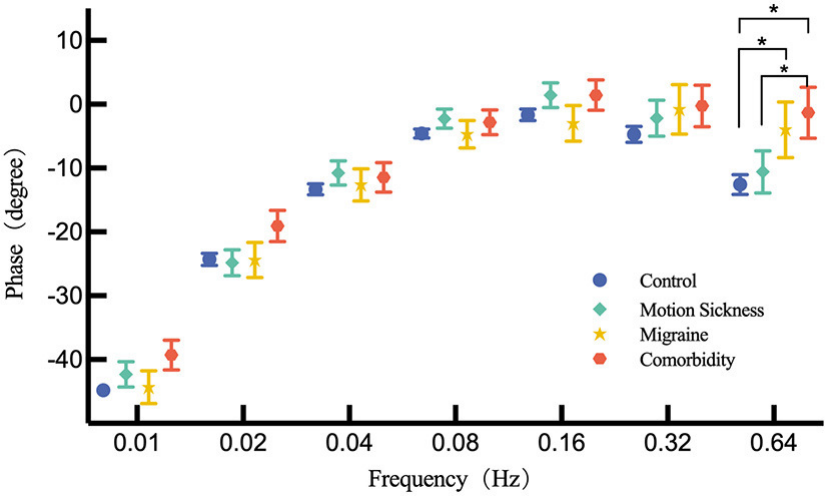
Comparison of the phase across MS group, migraine group, comorbidity group and control group for each frequency by multiple-factors ANOVA. Corrected phase values are presented by mean and standard error. The asterisk (*) indicates a significant difference exists.

When adjusted for the age, multiple-factors ANOVA indicated that no significant difference was observed among the four groups (motion sickness, migraine, comorbidity and control) at frequency from 0.01 to 0.32 Hz for phase. However, *Post-hoc* comparisons indicated that there was a statistically significant difference at 0.64 Hz for phase between migraine and control groups (*P* = 0.027). Phase lead of the migraine group is lower than that in the control group. Between the comorbidity and control groups, statistically significant difference in phase at 0.64 Hz exists (*P* = 0.003). Phase lead of comorbidity is lower than that in the control group. Significant difference for phase at 0.64 Hz was not observed between MS group and control group whereas was found between MS group and comorbidity group (*P* = 0.041). Likewise, phase lead of comorbidity group was lower than MS group and it was the lowest of the four groups. Figure 1 shows the phase difference of each frequency.

### VST and VS

Post-rotary gain and TC as parameters of VST and FI as a parameter of VS were analyzed. No significant age differences were found for the VST and VS (*P* > 0.05). For VS, our results indicated normative values for FI were <52.0% on the right side and <47.2% on the left side. Table 4 presents the normative data of VST in the control group and comparison across the MS, migraine, comorbidity and control group. The results indicated no significant differences exists among the four groups (*P* > 0.05 for all comparisons). There was also no significant difference in VS for the four groups.

**Table 4:** Comparison for velocity step test for the control group, motion sickness group, migraine group, and comorbidity group.

	**Parameters**	**Control**	**Motion sickness**	**Migraine**	**Comorbidity**	***P***
CW	Post-rotary gain	0.63 ± 0.13	0.57 ± 0.18	0.68 ± 0.12	0.73 ± 0.18	0.060
	TC	14.33 ± 3.96	14.51 ± 5.06	14.15 ± 3.73	14.90 ± 4.96	0.976
CCW	Post-rotary gain	0.62 ± 0.15	0.57 ± 0.14	0.62 ± 0.10	0.69 ± 0.12	0.317
	TC	14.85 ± 3.75	12.10 ± 4.21	14.39 ± 4.85	14.46 ± 4.32	0.330

*MS, motion sickness; CW, clock-wise; CCW, counter clock-wise; TC, time constant*.

## Discussion

The rotational chair is a physiological way to evaluate horizontal semicircular canal function. In this study, we sought to understand if the parameters were age-related and to build a normative dataset for different age groups. Our study shows that the difference of age groups for gain only exists at 0.01 Hz of SHAT. Moreover, it is positively related to age at 0.01 Hz even though the correlation coefficient was moderate. During the rotation, the most nauseating frequency is 0.1 Hz because it lasts the longest time and is a big challenge to keep alert, which explains why the gain values of lower frequency are less stable.

Moreover, the subjects' age range is 20–60 years old, and not equal distribution of subjects is in each group. So far, the relationship between VOR and age has been controversial. In some studies, the gain is relatively stable until an older age and is hardly affected by aging (15–18). In contrast, Chan et al. demonstrated VOR gain differences with age for the rotational chair test. They reported that the gain of SHAT across all the frequencies was inversely correlated to age (4). However, McGarvie et al. demonstrated that gains have a slightly negative relationship with age from the sixth-decade (16).

Similarly, Kim and Kim reported that the gain declined as age increased over 70 years for horizontal semicircular canal (17). Likewise, our data suggests gain varies significantly with age at 0.01 Hz under 60 years, which is inconsistent with the age range of the above studies. Gain is susceptible to the subjects' nervousness and alertness (19). In our study, we tried to ensure the subjects were constantly alert throughout the testing procedure.

Generally, when the head is rotated, the eyes move in the opposite direction with the same speed and angle to keep the visual steady. But they are not always at the same time and have a time delay. Phase highly corresponded to TC of VOR is measured to reflect the timing relationship between head movement and eye movement. The phase is a more reliable variable than the gain because it's steady and reproducible (20). This study indicated that phase angle decreased from phase leading to phase lag as the frequency increases in normal control. Our data also showed differences in phase existing from 0.08 to 0.64 Hz for all age groups, and phase lead values was found to be correlated with the age from 0.16 to 0.64 Hz. It may be linked to the velocity storage time constant. Previous studies demonstrated that age-dependent changes in the human VOR, especially decreasing time constant, which was explained by Bayesian optimal adaptation in the velocity storage occurring in response to the death of motion-sensing hair cells (21, 22). Asymmetry involves a comparison between the SPV to the right and leftward, indicating skewness within the peripheral vestibular system, which is often seen in unilateral peripheral vestibular lesion cases.

The second paradigm of the rotational chair test is VST. Gain and TC are the main parameters. Like SHAT, the gain of VST is associated with TC to explain vestibular functionality. To reduce the equipment wastage rate, post-rotatory TC is measured in our laboratory instead of per-rotatory. The stimulated semicircular canal is the opposite side of the rotation direction. A mathematical model has been proposed between TC of VST and phase of SHAT (23). As the phase lead increases, TC decreases. Though both reflect whether the VSM is normal or not, TC can be used to localize a lesion while phase not.

The last paradigm of the rotational chair test is VS. Differ from SHAT and VST, failure responses of VS usually suggest a lesion of central origin. The abnormal range of the Fixation Index is generally thought to be cerebellar involved. Lotfi et al. reported that children with attention deficit and hyperactivity disorder showed higher VOR gain but lower fixation abilities due to a lesion involving the middle cerebellum (24). Our study finds no correlation between age and parameters for VST and VS, indicating that both are steady and not affected by age.

The prolongation of an afferent signal existing VOR responses is achieved by velocity storage mechanism (VSM) (11, 12). In other words, VSM regulates the phase of the VOR system. The value of phase is influenced by the peripheral vestibular system and central nervous system. Peripheral dysfunction with pathologic damage results in abnormal information input to VSM, so phase lead may be beyond the normal range. Brainstem lesions may generate phase abnormally given that VSM is located in the brainstem. The pathological mechanism of MS is closely related to VSM by delaying the signals from the peripheral vestibule to the medial vestibular nucleus (25, 26). We expected differences in rotational chair test parameters between healthy groups and individuals with MS. The Barany Society presented diagnostic criteria for MS, including the observable signs or symptoms of gastrointestinal disturbance and thermoregulatory disruption, dizziness, and headache during exposure to physical motion (27). Clement and Reschke found no correlation between VOR gain and level of MS; however, they found that the lower the phase lead, the more severe the MS (14).

Previous literature showed the severity of symptoms was not related to the intensity of physical motion but impacted by stimulus frequency in MS susceptible individuals, which was usually triggered by low-frequency vertical, angular, and rotation motion (28). Irmak et al. used sinusoidal fore-aft motions to determine frequency responses among individuals with MS (29). They found individual variability in motion frequency sensitivity but no significant effect at the group level. In the present study, we found no significant differences between MS group and control group. This may be due to the fact that level of MS is mild. However, phase lead of comorbidity group which have both motion sickness and migraine was significantly lower than that of the control group. Moreover, some articles indicated that migraine was often associated with MS and enhanced symptoms with each other. They may share a common neural pathway involving the brainstem and serotonin modulation (9, 10). Therefore, the motion susceptibility of subjects with both motion sickness and migraine may be the most severe. But no significant difference was revealed in the TC of VST for comorbidity and control groups. Based on this finding, we speculate that phase is more sensitive to TC in assessing VSM.

It is reported that migraine is one of the leading causes of disability and affects people's life and work. The pathological theory of migraine has focused on the brainstem as a source of neurovascular disturbances (30). Evidence showed vestibular migraine patients were hypersensitive to self-motion and enhanced susceptibility to motion sickness during rotation (31). Murdin et al. demonstrated that both VM and migraine increased motion sickness susceptibility and were no different when assessed by Sickness Rating and short from Motion Sickness Susceptibility Questionnaire (32). However, how would migraineurs behave on chair rotary motions and whether they present differences in objective parameters remain unknown. We investigated motion sickness susceptibility differences objectively by measuring phase and TC. We found a significant difference in phase lead 0.64 Hz between the migraine and control groups. A series of studies have shown that velocity storage of semicircular canal signals is a critical factor for susceptibility to motion sickness. Receiving motion stimulation and having decayed symptoms is a prolonged period for individuals with motion sickness susceptibility. Longer time constants of the vestibular velocity store have been suggested to correlate with heighten motion sickness susceptibility (7, 33). So, it's not hard to understand that phase lead is lower for them.

But interestingly, no significant difference in TC of VST was observed. Our data supported that migraine is generally associated with high susceptibility during rotating motions, especially at higher frequencies. Unlike many previous studies, this study involved participants' susceptibility to motion, indicating SHAT is more sensitive to VST though they both reflect the performances of VSM.

## Study limitation

The study is limited to a single-center study. Due to a lack of volunteers, we have not established the normative values for younger than 20 and older than 60 years old. The sample size of MS, migraine and comorbidity groups is limited and age distribution was uneven. Future research should be undertaken to build a more comprehensive normative range.

## Conclusion

A small effect of age exists in SHAT. Gain is more susceptible to age at low frequency, while the phase is the opposite. No age difference exists in VST and VS. Subjects with both migraine and motion sickness show abnormal VSM and their motion susceptibility may be the most severe. When assessing their vestibular function with the rotational chair test, we should consider the bias of phase. According to our results, SHAT is more sensitive to VST in terms of reflecting motion susceptibility.

## Data availability statement

The original contributions presented in the study are included in the article/Supplementary material, further inquiries can be directed to the corresponding authors.

## Ethics statement

The studies involving human participants were reviewed and approved by Institute Review Board of Fudan University Eye Ear Nose and Throat Hospital (Reference Number 2020087). The patients/participants provided their written informed consent to participate in this study.

## Author contributions

WL and PW designed the study. JY and YW performed the study and wrote the manuscript. JZ and RH collected and analyzed the data for the study. All authors contributed to the article and approved the submitted version.

## Funding

This work was supported by the National Key R&D Program of China (No. 2022ZD0205400), the grant from the Science and Technology Commission of Shanghai (19441917100 and 20S31907000), the National Natural Science Foundation of China (Nos. 81922018 and 81771011), and the Three-year Action Plan of Shanghai Shenkang Hospital Development Center to Promote Clinical Skills and Clinical Innovation in Municipal Hospitals (SHDC2020CR3050B).

## Conflict of interest

The authors declare that the research was conducted in the absence of any commercial or financial relationships that could be construed as a potential conflict of interest.

## Publisher's note

All claims expressed in this article are solely those of the authors and do not necessarily represent those of their affiliated organizations, or those of the publisher, the editors and the reviewers. Any product that may be evaluated in this article, or claim that may be made by its manufacturer, is not guaranteed or endorsed by the publisher.
