# Supplementary material for: Normative data for rotational chair considering motion susceptibility
Source: Front Neurol. 2022 Aug 22;13:978442. doi: 10.3389/fneur.2022.978442 (PMC9441918; doi:10.3389/fneur.2022.978442)
Supplement: Supplementary file 1 [file Data_Sheet_1.docx]

**Questionnaire screening for Motion Sickness**

**Name**: ________. **Date of birth**: _________. **Gender:** ___________.

| 1 | Do you usually have motion sickness? | Yes | No |
| --- | --- | --- | --- |
| 2 | Do you usually feel sick by cars/buses/ships? | Yes | No |
| 3 | Do you usually feel sick when you play swing or roller coaster? | Yes | No |
| 4 | Did you also feel sick doing above things when you were as a child? | Yes | No |

The number 1 to 4 questions are about motion sickness. If subjects choose Yes in 3 out 4 questions, he/she is considered as motion sickness.
